# Disparities and protective factors in pandemic-related mental health outcomes: a Louisiana-based study

**DOI:** 10.3389/fpubh.2024.1404897

**Published:** 2024-08-08

**Authors:** Ariane L. Rung, Evrim Oral, Tyler Prusisz, Edward S. Peters

**Affiliations:** ^1^Department of Epidemiology, College of Public Health, University of Nebraska Medical Center, Omaha, NE, United States; ^2^Biostatistics and Data Science Program, School of Public Health, Louisiana State University Health Sciences Center, New Orleans, LA, United States; ^3^Epidemiology and Population Health Program, School of Public Health, Louisiana State University Health Sciences Center, New Orleans, LA, United States

**Keywords:** mental health, COVID-19, social support, resilience, social capital, social cohesion, race

## Abstract

**Introduction:**

The COVID-19 pandemic has had a wide-ranging impact on mental health. Diverse populations experienced the pandemic differently, highlighting pre-existing inequalities and creating new challenges in recovery. Understanding the effects across diverse populations and identifying protective factors is crucial for guiding future pandemic preparedness. The objectives of this study were to (1) describe the specific COVID-19-related impacts associated with general well-being, (2) identify protective factors associated with better mental health outcomes, and (3) assess racial disparities in pandemic impact and protective factors.

**Methods:**

A cross-sectional survey of Louisiana residents was conducted in summer 2020, yielding a sample of 986 Black and White adults. The exposure was overall pandemic impact, measured using the Epidemic-Pandemic Impacts Inventory, and the outcome was general well-being (GWB), measured with the General Well-Being Schedule. Potential protective factors included social support, resilience, and social cohesion. Linear regression models were constructed to examine the association between pandemic impact and GWB, with each protective factor added as an effect modifier. These relationships were further assessed for differences by race.

**Results:**

Pandemic stressors can be grouped into social, health, work, finance, and family-related impacts. Black persons displayed higher levels of pandemic impact as well as lower levels of social support, resilience, and social cohesion (*p* < 0.0001), highlighting existing racial disparities, though Black respondents and White respondents exhibited no differences in general-well being. Social support, resilience, and social cohesion were identified as protective factors for both groups (*p* < 0.0001, respectively), but these protective effects deteriorated as pandemic impacts increased. The addition of a pandemic impact by race interaction term was also significant in each model (*p* = 0.0020, *p* < 0.0001, and *p* = 0.0095, respectively) and showed that the protective effects of social support and resilience deteriorated more rapidly for Black persons than White persons, while the protective effects of social cohesion deteriorated more rapidly for White persons than Black persons.

**Discussion:**

This study emphasizes the importance of psychosocial resources in buffering the mental health impact of pandemics. It also suggests greater vulnerability for marginalized communities lacking access to crucial support systems. Findings underscore the need for targeted interventions that bolster access to social support, promote resilience, and strengthen social cohesion, particularly within minority groups. Additionally, policymakers should consider proactive measures to assist in recovery and mitigate the disproportionate impact of future crises on vulnerable populations.

## 1 Introduction

Beginning in March 2020, the COVID-19 pandemic caused major disruptions to society, including over 100 million confirmed cases and over 1 million deaths across the United States alone (1). Mitigation measures such as lockdowns, isolation and quarantine, physical distancing requirements, school closures, and restrictions on gatherings have all impacted citizens’ lives in unprecedented ways. Given the likelihood of future pandemics that may require similar mitigation measures (2), an understanding of their impact on mental health is critical. Research has detailed a range of mental health effects triggered by the pandemic. For example, a review of 68 studies found a significantly higher prevalence of depression, anxiety, insomnia, PTSD, and psychological distress in pandemic-affected countries around the world compared to rates usually observed in the general population (3). An October 2021 poll found that half of all US households reported a member experiencing serious problems with depression, anxiety, stress, or sleeping in the previous few months (4).

The psychological sequelae from the COVID-19 pandemic likely stem from a variety of stressors, including fear of becoming infected with and dying from COVID-19, loneliness and social isolation due to sheltering in place and other containment measures, financial hardship, and loss of employment, to name just a few (5–7). In 2021, 38% of households across the US reported facing serious financial problems in the previous months, 20% reported experiencing serious problems with getting childcare, and 24% reported having a worse job situation now compared to before the outbreak (4).

Long-term research priorities emphasize the need to better understand the buffering effects of social relationships during stressful events (8). Given the global impact that the pandemic has had on population mental health, identifying potential mitigating factors of these stressors on mental health will be critical. Social support is one such mitigating factor, as it is known to be protective for mental health and psychological well-being (9, 10). We define social support as the perceived availability of functional support in the form of emotional, instrumental, appraisal, and informational support (11, 12). In the context of acute disasters, social support can moderate the effects of disaster-related stressors on psychological outcomes (13). When levels of social support are high, they can be protective against poor mental health outcomes. It is thus reasonable to hypothesize that social support may ameliorate the impact of COVID-19 on individuals’ mental health. Indeed, direct evidence exists showing that the availability of social support was inversely correlated with anxiety level among Chinese college students during the early part of the pandemic (14).

Another potential mitigating factor is resilience, frequently defined as the ability to adapt well in the face of adversity, or to “bounce back” (15). It is often cited as a reason for why people who experience a traumatic event do not develop psychopathology (16). It has been well-studied in the disaster literature, where findings suggest that resilience characteristics can identify those who are exposed to a disruptive event but are still able to maintain a relatively healthy level of psychological functioning (16). A survey of US adults during the early pandemic found that psychological resilience was significantly lower compared to published norms, and lower scores were associated with worse mental health outcomes, such as depression, suicidal ideation, and anxiety (17). Resilience is thus another factor that could play a role in how individuals respond to the pandemic.

A third potential mitigating factor is social cohesion, which is the degree of trust, familiarity, values, and neighborhood network ties shared among residents (18). It is often construed as the foundation from which social capital arises and is distinct from social support in that it describes patterns of social interaction rather than an individual’s access to a single resource (18). Scholars have suggested that localities with higher levels of social capital may be better able to respond to the COVID-19 pandemic (19, 20). The feelings of trust, norms, and networks inherent in social cohesion/social capital may be particularly relevant during a pandemic, when these characteristics could facilitate collective action (19). They may benefit members of a community through the creation of cultures of obligation or expected reciprocity, enhanced community-based information channels, or the establishment of informal codes of socially normative behaviors (21). A number of studies have demonstrated that places with more social capital have fewer COVID-19 cases and deaths. For example, one study showed that an increase from the 25th to the 75th percentile of social capital in a US county would lead to an 18% decline in the cumulative number of COVID-19 infections and a 6% decline in the number of deaths (19). Research has also demonstrated that states with higher levels of social capital and social trust tend to have higher COVID testing rates, even after controlling for party affiliation, income, income inequality, and racial diversity (22). Certain forms of social capital have even been associated with better adherence to social distancing, as measured by the percentage of mobile devices that did not leave home in a given county on a given day (23). Given the evidence that social capital may also be inversely associated with common mental disorders (24), it is possible that social capital plays a role in softening the effects of COVID-related stressors on mental health.

Race complicates efforts to disentangle the pandemic’s effects on mental health. Black persons in the United States were disproportionately affected by COVID-19 compared to other races. They had a higher COVID-19 mortality rate compared to White persons (88/100,000 vs. 40/100,000, respectively) and were twice as likely to be hospitalized and 3.6 times as likely to die from COVID-19 compared to White persons (25). These disparities appeared to be driven primarily by systemic structural disadvantages, such as greater barriers to educational attainment, lower household income, and residing in lower income neighborhoods (26), all of which predisposed them to chronic diseases, which in turn lead to development of comorbidities that put them at higher risk for COVID-19. A direct path to COVID-19 infection also existed, as evidenced by their disproportionate representation in essential or high risk jobs that prevented them from sheltering in place, as well as crowded housing that facilitated transmission, and an increased likelihood of living with health care workers (25, 27). At the same time, Black persons were experiencing collective trauma as a result of police killings of figures such as George Floyd and Breonna Taylor, resulting in independent impacts on their mental health (28, 29), and making it even more difficult to unravel the mental health effects of the pandemic.

In addition to these pandemic- and context-related impacts, Black individuals already tend to bear a greater burden from unmet mental health needs. While the prevalence of certain mental disorders among Black individuals is not generally higher compared to White individuals, they are less likely to receive diagnoses and treatment, particularly high-quality treatment, for their mental health concerns (7, 30). Moreover, psychological effects from the pandemic were more pronounced among racial and ethnic minorities, as evidenced by elevated suicide rates, suicidal ideation, grief, and related mental health symptoms for family members of those who died due to COVID-19 (27).

Potential mitigating factors such as social support, resilience, and social cohesion may operate differently for Black persons compared to White persons. While evidence suggests that racial minorities in the US received less social support from family, friends, and partners during the pandemic compared to White persons (31), the literature on racial/ethnic differences in resilience is mixed; some studies demonstrate higher levels of resilience among Black people (32), while others show a lower prevalence (33) or no difference (34). An emerging perspective on resilience in the Black community emphasizes a greater focus on the strength of Black people, who have collectively survived generations of trauma; examples of extraordinary strength of mind and body under such adverse conditions suggest that resilience may be an overlooked target of intervention in this population (35). The literature on racial/ethnic differences in individual-level social cohesion is even more sparse, although there are some indications that Black persons score lower on social cohesion scales than White persons (36). These potential differences in how mitigating factors operate underscore the need to examine the role of racial disparities in the promotion of pandemic-related psychological well-being in order to identify more targeted pandemic responses.

The objectives of this study are to (1) describe the specific COVID-19-related impacts associated with general well-being, (2) identify protective factors associated with better mental health outcomes, and (3) assess racial disparities in pandemic impact and protective factors.

## 2 Materials and methods

### 2.1 Participants and survey administration

Data were collected using a cross-sectional survey of Louisiana residents between July 23 and September 6, 2020. Surveys were administrated through Qualtrics XM, a commercial survey sampling and administration company. Participants were acquired from existing pools of research panel samples who had agreed to be contacted for research studies. Individuals received an email invitation to the survey if they were preregistered for Qualtrics Panels and had completed an online baseline proprietary survey. Panelists were then invited to participate electronically and opted in by activating a survey link directing them to the study consent webpage and survey instrument. After consenting, participants were directed to the questionnaire. The LSUHSC-New Orleans Institutional Review Board reviewed and exempted the study.

To ensure the representativeness of the survey sample, Qualtrics strived to balance participants by age, gender, and race distributions for the state of Louisiana. In order to participate, respondents had to reside in Louisiana and be over the age of 18 years. 1,050 participants completed the survey. To ensure sufficient sample sizes by race, 986 respondents who self-reported White or Black race were retained in the final analytic dataset.

### 2.2 Measures

#### 2.2.1 Outcome

The outcome for this study was general well-being, which measures subjective feelings of psychological well-being and distress. The General Well-Being Schedule (GWB) is a brief, reliable, valid, and widely used self-administered questionnaire that addresses subjective well-being, using 18 items developed for the U.S. Health and Nutrition Examination Survey (37, 38). Each item incorporates the time frame “during the last month,” with lower scores reflecting distress and higher scores reflecting positive well-being. The possible range of scores is 0–110. While the measure has established cut-offs, with 0–60 reflecting “severe distress,” 61–72 “moderate distress,” and 73–110 “positive well-being,” GWB is treated as a continuous variable for this analysis.

#### 2.2.2 Exposure of interest

The primary exposure for this study was negative pandemic-related impacts. A series of questions from the Epidemic-Pandemic Impacts Inventory (EPII) (39) was asked about respondents’ experiences since the beginning of the pandemic in Louisiana in March 2020, including items such as being laid off from a job, being unable to pay bills, being unable to access medical care, or having increased conflict with a partner. Possible response options were: no one in household affected (0), one person in household affected (1), and at least 2 people in household affected (2). Responses to the questions were subjected to a principal components analysis (40). The principal axis method was used to extract the components, and this was followed by a varimax (orthogonal) rotation. Variables that did not load on any factors or that loaded on more than one component were excluded, resulting in a final selection of 23 items. Only the first five components were retained for rotation, which accounted for 54% of the total variance. Questionnaire items and corresponding factor loadings are presented in Table 1. In interpreting the rotated factor pattern, an item was said to load on a given component if the factor loading was 0.40 or greater for that component and was less than 0.40 for the other. Scores were summed to create a single overall pandemic impact score. Higher scores are indicative of higher levels of negative pandemic-related impact.

**Table 1:** Rotated factor pattern and final communality estimates from principal component analysis of pandemic-related impacts (*N* = 986).

	Component										
Item	1		2		3		4		5		Communality estimate
Laid off from job	9		-1		66	*	2		34		0.56
Had to close own business	-2		15		67	*	20		2		0.52
Reduced work hours or furloughed	18		8		68	*	5		16		0.53
Had to lay-off or furlough employees or people supervised	−4		23		67	*	25		4		0.57
Hard time making the transition to working from home	7		21		49	*	37		−2		0.42
Unable to get enough food	6		21		13		16		82	*	0.76
Unable to get healthy food	8		25		6		22		78	*	0.72
Unable to pay important bills like rent or utilities	8		12		27		15		73	*	0.64
Unable to get needed medications	7		53	*	21		36		26		0.52
Childcare or babysitting unavailable when needed	9		15		28		59	*	13		0.48
More conflict with child or children	17		4		7		80	*	12		0.68
Family or friends had to move into your home	−5		19		30		61	*	14		0.52
Increase in conflict with a partner or spouse	24		11		8		54	*	18		0.40
Separated from family or close friends	64	*	17		−3		6		6		0.45
Unable to do enjoyable activities or hobbies	74	*	9		5		7		11		0.58
Unable to visit loves ones in hospital	45	*	39		17		3		11		0.40
Family celebrations canceled/restricted	79	*	5		1		8		−1		0.63
Planned travel/vacations canceled	70	*	6		12		7		−9		0.53
Increase in health probs not related to pandemic	14		64	*	6		19		22		0.52
Eating more unhealthy foods	50	*	13		2		23		25		0.38
Unable to access medical care for serious condition	−3		68	*	20		27		9		0.59
Delay getting medical care	21		72	*	7		2		5		0.57
Isolated due to existing health conditions that increase risk of infection	21		65	*	13		0		15		0.50

Values are multiplied by 100 and rounded to nearest integer. *Indicates absolute values greater than or equal to 0.4.

Scores were also summed to create the five components of pandemic-related impacts for descriptive purposes. They were identified as: social impacts, health impacts, work impacts, financial impacts, and family impacts. Social impacts related to the pandemic included being unable to do enjoyable activities or having family celebrations canceled. Health impacts included isolation due to existing health conditions or delays in getting medical care. Work impacts included having reduced work hours or being laid off from a job. Finance impacts included being unable to pay bills or get enough food. Family impacts included increases in conflict with partners or children and childcare availability.

#### 2.2.3 Effect modifiers

Three variables were hypothesized as potential buffers of the pandemic impact-general well-being relationship and treated as possible effect modifiers.

Social support, defined as the perceived availability of functional support in the form of emotional, instrumental, appraisal, and informational support, was measured using the 19-item MOS Social Support Survey (12). Scores for an overall index were calculated by averaging the scores for all items, resulting in a range of 1 to 5, with higher scores indicating more support. Scores were then dichotomized at the median into low and high support.

Resilience, a measure of successful stress-coping ability, was assessed using the Connor-Davidson Resilience Scale 10 (CD-RISC-10), which reflects the ability to bounce back from a variety of challenges that can arise in life (41). The measure consists of 10 items rated on a scale of 0 (not true at all) to 4 (true nearly all the time). Example items include “I am able to adapt when changes occur” and “I can deal with whatever comes my way.” An overall score was calculated by summing the 10 items, resulting in a range of 0 to 40, with higher scores suggesting greater resilience. Scores were then dichotomized at the median into low and high resilience.

The third potential buffer was social cohesion, a component of social capital (42). Social cohesion is conceptualized as the degree of trust, familiarity, values, and neighborhood network ties shared among residents and is measured at the individual level. Five items (42) asked respondents how strongly they agreed with the following statements: “This is a close-knit neighborhood” (reverse coded), “People around here are willing to help their neighbors” (reverse coded), “People in my neighborhood generally do not get along with each other,” “People in my neighborhood do not share the same values,” and “People in my neighborhood can be trusted” (reverse coded). Response options ranged from strongly agree (1) to strongly disagree (5). A score for social cohesion was calculated by averaging items, resulting in a range of 1 to 5, with higher scores reflecting more social cohesion. Scores were then dichotomized at the median into low and high cohesion.

#### 2.2.4 Other covariates

Participants self-reported their race; only those self-reporting as White or Black/African American were retained for this analysis. Other variables included sex (male vs. female), age (18–24 years, 25–44 years, 45–64 years, and 65+ years), marital status (married or partnered vs. widowed, divorced, separated, or single), income (less than $50,000/year vs. $50, 000/year or more), and presence of children 0–17 years in household.

### 2.3 Analysis

Descriptive statistics were calculated for demographic variables including race, the general well-being outcome, pandemic experiences (overall score as well as subscores), and the potential buffering characteristics of social support, resilience, and social cohesion. They are presented for the whole sample and then stratified by race. *p*-values for racial differences were calculated using chi-square tests for categorical variables and t-tests for continuous variables. Simple linear regression modeling was performed to assess the unadjusted associations between each demographic characteristic, pandemic-related impact, and buffering characteristic with general well-being. Subsequently, three separate multiple linear regression models were used to assess the effects of race and buffering characteristics along with their interactions with pandemic-related impacts (overall score only) on general well-being. These models were adjusted for potential confounding variables of sex, age, marital status, income, and presence of children in household. All statistical analyses were performed using SAS 9.4 for Windows (SAS Institute Inc., Cary, North Carolina).

## 3 Results

Table 2 presents the participant characteristics for the total sample by race. 68% of the sample is White and 32% Black. The sample is predominantly female, 25–44 years, and evenly split between married and single. 57% of respondents had a 2019 household income of less than $50,000 per year. The mean general well-being score was 65.14 (SD 21.3). About half of respondents reported having high levels of social support, 45% reported high levels of resilience, and 44% reported high levels of social cohesion. Black respondents were more likely than White respondents to be female, younger than 44 years, single, have a household income less than $50,000 per year, and to have any children under 18 years in the household. Black persons were more likely than White persons to report having low levels of all three buffering characteristics of social support, resilience, and social cohesion.

**Table 2:** Demographics of sample, Louisiana, July–September 2020, *N* = 986.

	Total		White (*n* = 673)		Black (*n* = 313)		*p*-value for race differences
	N	%	N	%	N	%	
General Well-Being (mean, SD)	65.14	21.31	65.46	22.31	64.45	18.99	0.4637
Sex							0.0027
Male	394	40.08	290	43.28	104	33.23	
Female	589	59.92	380	56.72	209	66.77	
Age							<0.0001
18–24 yrs	192	19.47	81	12.04	111	35.46	
25–44 yrs	360	36.51	240	35.66	120	38.34	
45–64 yrs	269	27.28	209	31.05	60	19.17	
65+ yrs	165	16.73	143	21.25	22	7.03	
Marital Status							<0.0001
Married/partnered	470	48.11	386	57.53	84	27.45	
Single	507	51.89	285	42.47	222	72.55	
Income, Annual HH, 2019							<0.0001
Less than $50 K/yr	558	56.59	335	49.78	223	71.25	
$50 K/yr or more	428	43.41	338	50.22	90	28.75	
Any children 0–17 years in HH							<0.0001
No	613	62.17	449	66.72	164	52.40	
Yes	373	37.83	224	33.28	149	47.60	
Social support score							<0.0001
Low	495	50.20	303	45.02	192	61.34	
High	491	49.80	370	54.98	121	38.66	
Resilience score							0.0463
Low	543	55.07	356	52.90	187	59.74	
High	443	44.93	317	47.10	126	40.26	
Social cohesion score							<0.0001
Low	487	55.98	307	50.58	180	68.44	
High	383	44.02	300	49.52	83	31.56	

Missing values were reported for gender (*n* = 3), marital status (*n* = 9), and social cohesion score (*n* = 116).

Pandemic-related impacts are listed in Table 3 and grouped by subscore category. Overall pandemic impact scores ranged from 0 to 39, with a mean of 10.0 (SD 9.6). Within the sample, 95% scored 24 or lower on the overall scale; 75% scored under 14 or lower, 50% scored 9 or lower, and 10% of the sample scored 2 or lower. Compared to White persons, Black persons tended to experience higher levels of pandemic-related impacts, particularly in the categories of work (White persons mean 1.07, SD 1.49 vs. Black persons mean 1.78 SD 2.10, *p* < 0.0001), finance (White persons mean 0.86, SD 1.60 vs. Black persons mean 1.44 SD 1.70, *p* < 0.0001), family (White persons mean 0.82, SD 1.43 vs. Black persons mean 1.09 SD 1.67, *p* = 0.0116), and the overall pandemic impact score (White persons mean 9.61, SD 6.89 vs. Black persons mean 10.94 SD 8.33, *p* = 0.0143).

**Table 3:** Pandemic-related impacts (*N* = 986).

	Total (*N* = 986)		White persons (*n* = 673)		Black persons (*n* = 313)		*p*-value
	Mean	SD	Mean	SD	Mean	SD	
Social impacts	5.22	3.29	5.34	3.28	4.95	3.28	0.0842
Separated from family or close friends	0.80	0.81	0.84	0.82	0.71	0.76	0.0226
Unable to do enjoyable activities or hobbies	1.06	0.79	1.08	0.81	1.01	0.76	0.1842
Unable to visit loved ones in hospital	0.57	0.77	0.54	0.77	0.62	0.75	0.1113
Family celebrations canceled/restricted	1.04	0.83	1.12	0.83	0.88	0.79	<0.0001
Planned travel/vacations canceled	0.94	0.85	0.96	0.86	0.89	0.81	0.2166
Eating more unhealthy foods	0.81	0.81	0.80	0.83	0.83	0.77	0.5714
Health impacts	1.57	2.10	1.52	2.00	1.67	2.32	0.3215
Increase in health problems not related to pandemic	0.31	0.59	0.32	0.59	0.30	0.59	0.6579
Unable to access medical care for serious condition	0.16	0.45	0.13	0.40	0.24	0.54	0.0013
Delay getting medical care	0.41	0.67	0.42	0.67	0.39	0.67	0.4614
Isolated due to existing health conditions that increase risk of infection	0.46	0.68	0.47	0.69	0.44	0.67	0.6039
Unable to get needed medications	0.23	0.55	0.19	0.51	0.31	0.61	0.0034
Work impacts	1.30	1.74	1.07	1.49	1.78	2.10	<0.0001
Laid off from job	0.31	0.54	0.25	0.50	0.43	0.60	<0.0001
Had to close own business	0.11	0.37	0.09	0.32	0.17	0.46	0.0055
Reduced work hours or furloughed	0.43	0.60	0.37	0.57	0.54	0.65	0.0002
Had to lay-off or furlough employees or people supervised	0.17	0.45	0.12	0.37	0.29	0.58	<0.0001
Hard time making the transition to working from home	0.28	0.54	0.24	0.51	0.36	0.60	0.0034
Finance impacts	1.05	1.65	0.86	1.60	1.44	1.70	<0.0001
Unable to get enough food	0.31	0.63	0.24	0.58	0.45	0.70	<0.0001
Unable to get healthy food	0.34	0.66	0.29	0.62	0.46	0.72	0.0003
Unable to pay important bills like rent or utilities	0.40	0.65	0.33	0.64	0.54	0.68	<0.0001
Family impacts	0.90	1.52	0.82	1.43	1.09	1.67	0.0116
Childcare or babysitting unavailable when needed	0.19	0.48	0.14	0.44	0.28	0.55	0.0001
More conflict with child or children	0.26	0.57	0.23	0.57	0.31	0.59	0.0422
Family or friends had to move into your home	0.13	0.41	0.10	0.37	0.19	0.47	0.0028
Increase in conflict with a partner or spouse	0.32	0.64	0.33	0.66	0.30	0.59	0.4658
**Overall pandemic impact**	**10.03**	**7.40**	**9.61**	**6.89**	**10.94**	**8.33**	**0.0143**

Unadjusted associations of participant characteristics with general well-being are shown in Table 4. Demographic characteristics associated with higher levels of general well-being include being male, over age 65 years, married or partnered, and having an income over $50,000/year. Those having children between the ages of 0 and 17 years in the house scored lower on the general well-being scale than their counterparts without children. The pandemic impact score was negatively related to general well-being; for every one-unit increase in the overall pandemic score, general well-being decreased by 1.19 (95% CI −1.35, −1.03). The three buffering characteristics were positively associated with general well-being. Individuals with high social support had an average general well-being score that was 15.21 points (95% CI 12.73, 17.70) higher than those with low social support. Similarly, those with high resilience had an average general well-being score 22.13 points (95% CI 19.85, 24.42) higher than those with low resilience, and those with high social cohesion were 11.84 points (95% CI 9.13, 14.56) higher than those with low social cohesion.

**Table 4:** Unadjusted correlates of general well-being.

	N	Estimate	Confidence limits		*p*-value
**Race**					
White race (vs. Black)	986	1.01	−1.85	3.86	0.4888
**Sex**					
Male sex (vs. female)	983	10.14	7.50	12.78	<0.0001
**Age**	986				
18–24 years (vs. 65+ years)		−19.25	−23.44	−15.07	<0.0001
25–44 years (vs. 65+ years)		−19.16	−22.87	−15.45	<0.0001
45–64 years (vs. 65+ years)		−12.46	−16.36	−8.55	<0.0001
**Marital status**	977				
Married/partnered (vs. single)		4.64	1.98	7.31	0.0006
**Income**					
Under $50 K/yr (vs. over $50 K/yr)	986	−7.35	−9.99	−4.71	<0.0001
**Children**					
Any children 0–17 yrs. in HH (vs. none)	986	−5.86	−8.58	−3.15	<0.0001
**Pandemic impacts**					
Overall pandemic score	986	−1.19	−1.35	−1.03	<0.0001
**Buffering characteristics**					
High social support (vs. low)	986	15.21	12.73	17.70	<0.0001
High resilience score (vs. low)	986	22.13	19.85	24.42	<0.0001
High social cohesion (vs. low)	870	11.84	9.13	14.56	<0.0001

**Figure 1:**
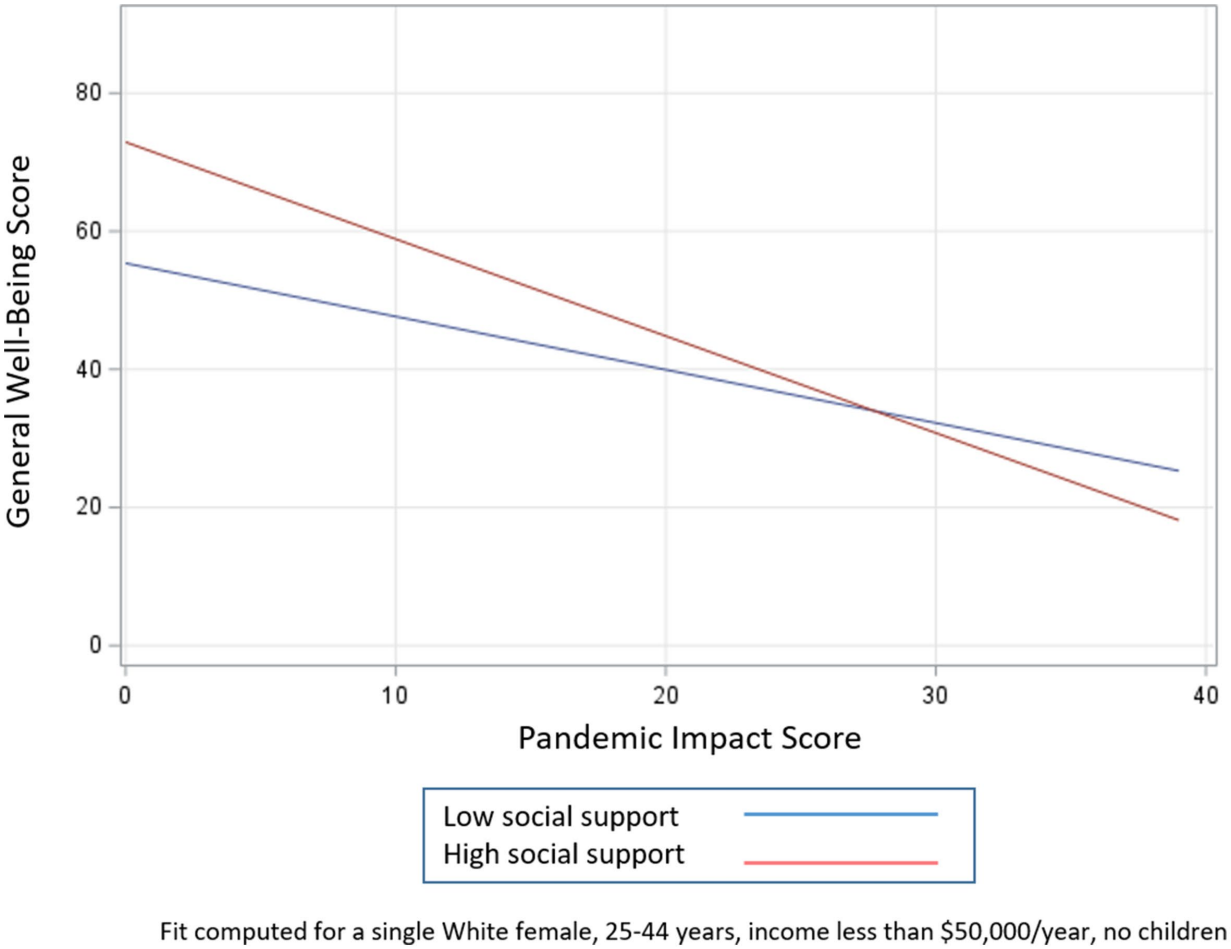
Relationship between pandemic impact and general well-being by levels of social support.

**Figure 2:**
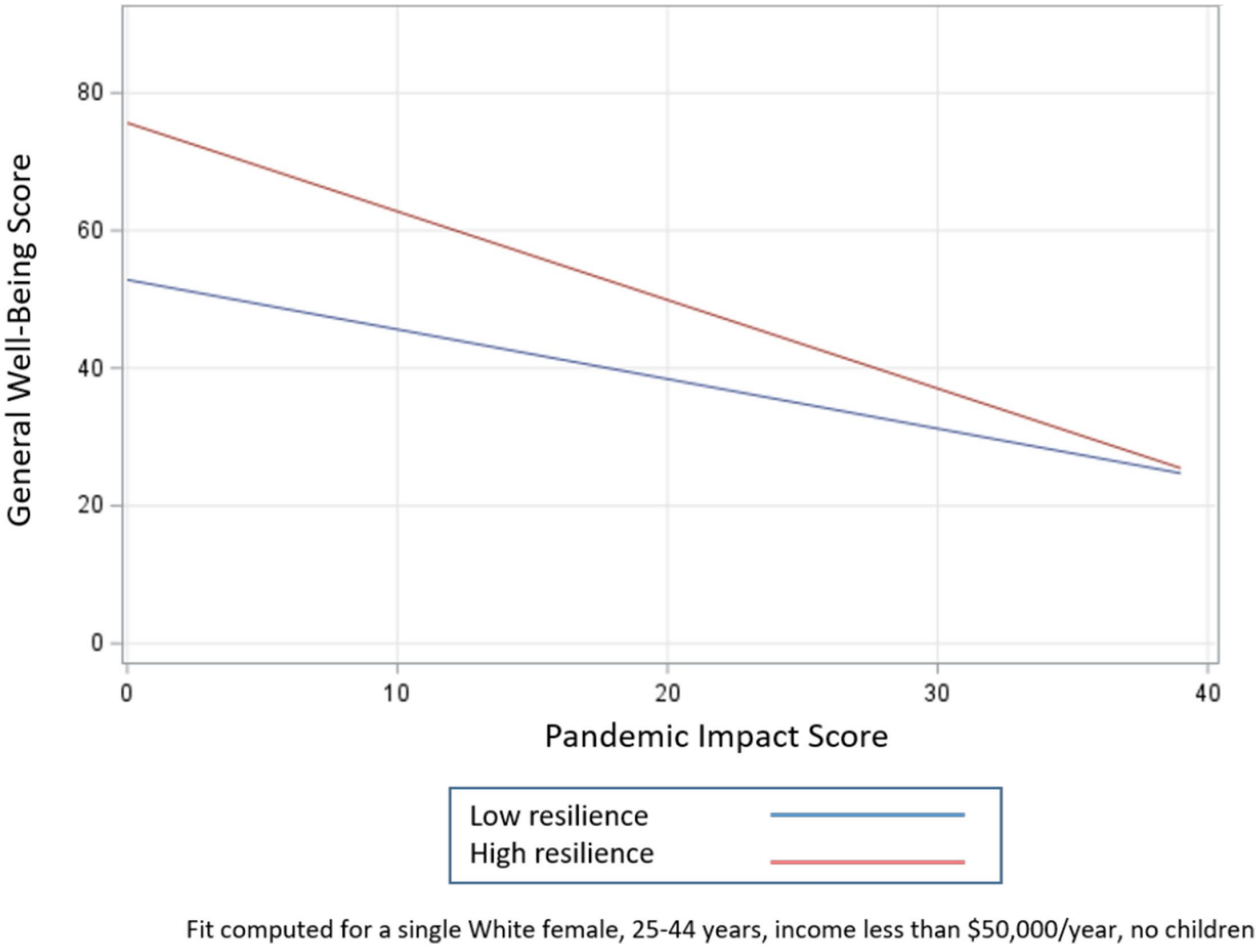
Relationship between pandemic impact and general well-being by levels of resilience.

**Figure 3:**
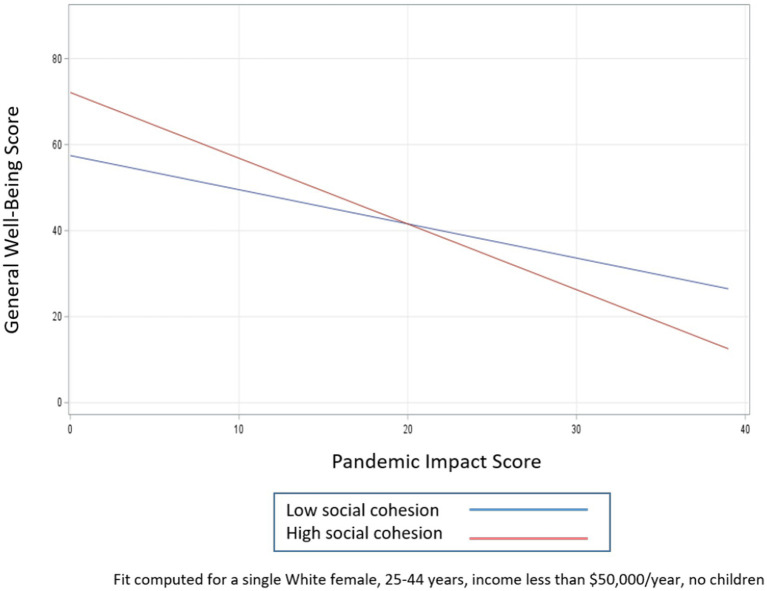
Relationship between pandemic impact and general well-being by levels of social cohesion.

To examine how the potential moderating effects of social support, resilience, and social cohesion influence the relationship between general well-being and pandemic impacts, we created three models that included an interaction term for each buffering characteristic, adjusting for race, sex, age, marital status, income, and presence of children (see Table 5; Figures 1–3). Respondents with higher levels of social support and social cohesion had higher levels of general well-being, holding race constant, until they reached extreme levels of pandemic impact (e.g., > 90–95th percentile), after which these factors ceased to be protective and actually became detrimental to well-being (pandemic impact by social support interaction coefficient = −0.63, 95% CI −0.93, −0.33 and pandemic impact by social cohesion interaction coefficient = −0.73, 95% CI −1.07, −0.40). For resilience, though, we found that respondents with higher levels of resilience had high levels of general well-being, a protective effect that endured regardless of level of pandemic impact (pandemic impact by resilience interaction coefficient = −0.57, 95% CI −0.85, −0.29).

**Table 5:** Adjusted effects of buffering characteristics on general well-being.

	N	Estimate	Confidence Limits		*p*-value
**Model A – Social Support**	974				
Overall pandemic impact score		−0.77	−0.96	−0.58	<0.0001
High social support (vs. low)		17.55	13.81	21.28	<0.0001
White race (vs. Black)		−5.71	−8.21	−3.21	<0.0001
Pandemic impact * social support		−0.63	−0.93	−0.33	<0.0001
**Model B – Resilience**	974				
Overall pandemic impact score		−0.72	−0.90	−0.54	<0.0001
High resilience (vs. low)		22.81	19.43	26.18	<0.0001
White race (vs. Black)		−4.52	−6.83	−2.20	0.0001
Pandemic impact * resilience		−0.57	−0.85	−0.29	<0.0001
**Model C – Social Cohesion**	862				
Overall pandemic impact score		−0.79	−0.99	−0.60	<0.0001
High social cohesion (vs. low)		14.66	10.60	18.73	<0.0001
White race (vs. Black)		−6.51	−9.27	−3.75	<0.0001
Pandemic impact * social cohesion		−0.73	−1.07	−0.40	<0.0001

All models adjusted for potential confounders of sex, age, marital status, income, and presence of children in the home.

**Figure 4:**
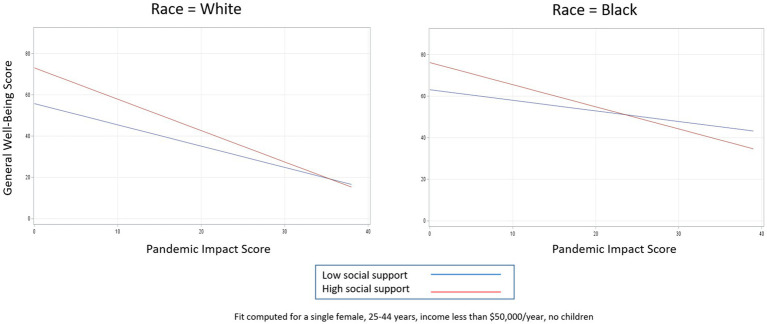
Relationship between pandemic impact and general well-being by levels of social support, stratified by race.

**Figure 5:**
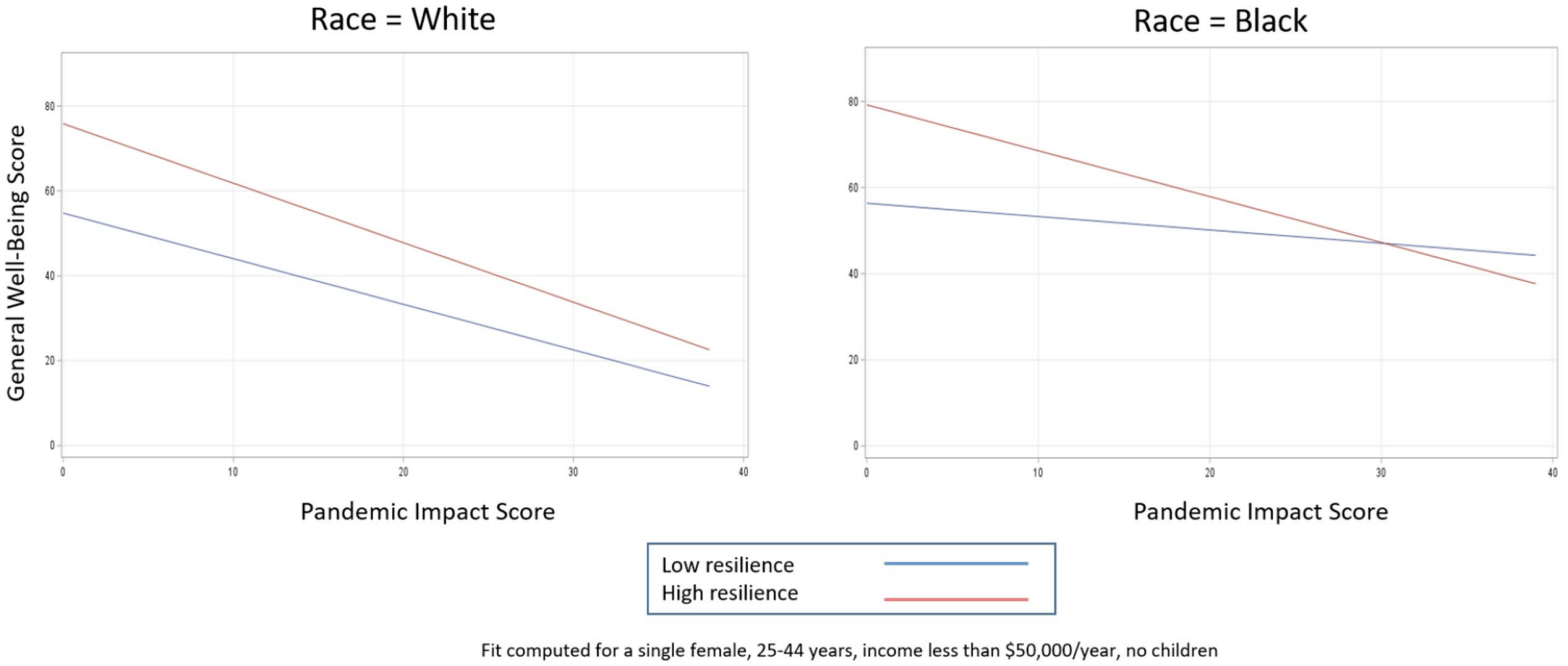
Relationship between pandemic impact and general well-being by levels of resilience, stratified by race.

**Figure 6:**
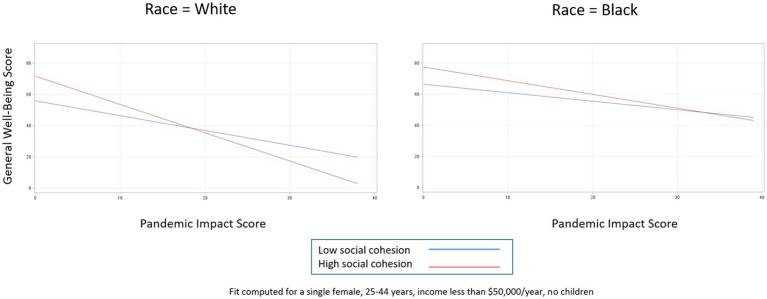
Relationship between pandemic impact and general well-being by levels of social cohesion, stratified by race.

To explore how race further impacts the general well-being—pandemic impact relationship, we then included a pandemic impact by race interaction term. Table 6 shows the adjusted effects of race and each potential buffering characteristic on general well-being in three separate models. In each of the models shown in Table 6, both interaction terms were significantly associated with general well-being, so for each buffer we present models stratified by race for simplicity of interpretation, along with their corresponding interaction plots (Table 7; Figures 4–6).

**Table 6:** Adjusted effects of buffering characteristics and race on general well-being.

	N	Estimate	Confidence Limits		*p*-value
**Model A – Social Support**	974				
Overall pandemic impact score		−0.53	−0.78	−0.29	<0.0001
High social support (vs. low)		16.39	12.60	20.18	<0.0001
White race (vs. Black)		−0.81	−4.79	3.17	0.6894
Pandemic impact * social support		−0.52	−0.82	−0.21	0.0011
Pandemic impact * race		−0.48	−0.78	−0.18	0.0020
**Model B – Resilience**	974				
Overall pandemic impact score		−0.43	−0.65	−0.20	0.0002
High resilience (vs. low)		21.94	18.57	25.31	<0.0001
White race (vs. Black)		1.30	−2.35	4.94	0.4860
Pandemic impact * resilience		−0.47	−0.75	−0.18	0.0012
Pandemic impact * race		−0.56	−0.84	−0.29	<0.0001
**Model C – Social Cohesion**	862				
Overall pandemic impact score		−0.57	−0.83	−0.30	<0.0001
High social cohesion (vs. low)		13.88	9.79	17.98	<0.0001
White race (vs. Black)		−1.96	−6.36	2.44	0.3825
Pandemic impact * social cohesion		−0.66	−1.00	−0.32	0.0001
Pandemic impact * race		−0.43	−0.75	−0.10	0.0095

All models adjusted for potential confounders of sex, age, marital status, income, and presence of children in the home.

**Table 7:** Adjusted effects of pandemic impact on general well-being by levels of buffering characteristics, stratified by race.

	Estimate	Confidence limits		*p*-value	Estimate	Confidence limits		*p*-value
	N				N			
	White persons				Black persons			
**Model A – Social Support**	668				306			
Overall pandemic impact score	−1.03	−1.30	−0.77	<0.0001	−0.51	−0.78	−0.24	0.0002
High social support (vs. low)	17.31	12.75	21.88	<0.0001	13.06	6.30	19.83	0.0002
Pandemic impact * social support	−0.49	−0.86	−0.12	0.0097	−0.55	−1.11	−0.003	0.0487
**Model B – Resilience**	668				306			
Overall pandemic impact score	−1.07	−1.31	−0.83	<0.0001	−0.31	−0.57	−0.05	0.0195
High resilience (vs. low)	21.07	16.98	25.16	<0.0001	22.83	16.88	28.79	<0.0001
Pandemic impact * resilience	−0.33	−0.68	0.02	0.0639	−0.75	−1.24	−0.27	0.0024
**Model C – Social Cohesion**	605				257			
Overall pandemic impact score	−0.95	−1.21	−0.68	<0.0001	−0.55	−0.83	−0.26	0.0002
High social cohesion (vs. low)	15.80	10.98	20.63	<0.0001	11.07	3.54	18.59	0.0039
Pandemic impact * social cohesion	−0.86	−1.27	−0.45	<0.0001	−0.33	−0.91	0.24	0.2523

All models adjusted for sex, age, marital status, income, and presence of children in the home.

Table 7 shows the effect of pandemic impact on general well-being by high versus low levels of each buffering characteristic, stratified by race. In the case of social support (Figure 4), for White persons, high levels of social support are protective for general well-being at almost every level of pandemic impact (pandemic impact by social support interaction coefficient = −0.49, 95% CI −0.86, −0.12). For Black persons, high levels of social support are protective for general well-being as well, but only while pandemic impact scores are below about the 95th percentile; once pandemic impact scores are in the extreme range (top 5th percentile), high levels of social support result in worse general well-being compared to low levels of social support (pandemic impact by social support interaction coefficient = −0. 55, 95% CI −1.11, −0.003). For both races, higher pandemic impact scores are associated with decreased general well-being as expected; however, having high social support further aggravates this decrease, and this phenomenon happens more quickly for Black persons than White persons.

In the case of resilience (Figure 5), for White persons, high levels of resilience are protective for general well-being at every level of pandemic impact, as indicated by the almost parallel lines and non-significant interaction term (pandemic impact by resilience interaction coefficient = −0.33, 95% CI −0.68, 0.02). For Black persons, high levels of resilience are similarly protective for general well-being but only until pandemic impact scores reach extreme levels (e.g., > 95th percentile), after which high levels of resilience result in worse general well-being compared to low levels of resilience (pandemic impact by resilience interaction coefficient = −0.75, 95% CI −1.24, −0.27).

For social cohesion (Figure 6), we see almost the opposite effects. For White persons, high levels of social cohesion are protective for general well-being until pandemic impact levels reach almost the 90th percentile (pandemic impact by social cohesion interaction coefficient = −0.86, 95% CI −1.27, −0.45). For Black persons, by contrast, high levels of social cohesion are protective for general well-being at almost all pandemic impact levels, as indicated by the non-significant interaction term (pandemic impact by social cohesion interaction coefficient = −0.33, 95% CI −0.91, 0.24).

## 4 Discussion

### 4.1 Pandemic impacts

The first objective of this study was to describe the specific COVID-19-related impacts associated with general well-being. *The overall pandemic score, consisting of pandemic experiences faced by respondents and their household members across multiple domains, was negatively related to general well-being, with the subscales of finance, family, health, and work particularly important in unadjusted associations*. These results suggest that general well-being was negatively associated with a variety of pandemic consequences quite apart from getting sick, which should be taken into account when addressing the mental health of the broader population. Research has shown vast mental health impacts related to the pandemic, but only a few studies have examined the relationship between specific aspects of the pandemic and mental health. One such example, a North American study of almost 2,500 adults, identified a variety of pandemic-related stressors, such as personal threat to health, social isolation, financial insecurity, occupational difficulty, and resource scarcity, and found them to be independently associated with depression at follow-up (43). The results of the current study corroborate these findings. Other studies have explored pandemic impacts in specific populations (44–49) but did not focus on mental health. The present study adds to the literature by describing how mental health is related not only to the overall pandemic impact, but to the specific areas of finance, family, health, and work impacts as well. These findings suggest potential targets for interventions related to addressing the psychosocial impacts of pandemics.

### 4.2 Buffering effects

The second objective of this study was to identify factors that may mitigate the effects of COVID-19-related impacts on general well-being. We first confirmed that *higher levels of social support, resilience, and social cohesion were all positively associated with general well-being*. In examining whether they were protective in the pandemic impact – general well-being relationship, we found that respondents with higher levels of social support and social cohesion had higher levels of general well-being, holding race constant, but only up to a certain point on the pandemic impact scale, after which these factors ceased to be protective and actually became detrimental to well-being. For resilience, though, we found that respondents with higher levels of resilience had high levels of general well-being, a protective effect that endured (albeit at increasingly smaller levels) regardless of level of pandemic impact.

#### 4.2.1 Application of social support deterioration theory

A possible explanation for this pattern of circumstances can be found in the social support deterioration theory (50), which posits that certain events, such as disasters, result in support *mobilization* that limits psychological distress. However, at the same time, disaster contributes to support *deterioration*, as resources that are initially mobilized are finite and tend to dissipate with time. For example, a community sample of adults was interviewed before and several times after a flood. Respondents had experienced different levels of physical, material, and personal losses as a result of the flood. Study findings supported the hypothesis that disaster victims experienced the impact of the flood both directly through immediate loss and exposure to trauma as well as indirectly through deterioration of their social supports. In other words, the negative effect of the stress (flood) was reflected in the weakening of the capability of support systems to guard against the disaster’s detrimental impact (50). It is likely that a similar phenomenon is occurring with the COVID-19 pandemic. The demands of the pandemic likely motivated individuals to activate their support networks and other resources. However, for individuals who experienced more intense pandemic impacts, any support that was initially protective in terms of mental health likely could not stand up to the deeper effects of the crisis. In this case, the pandemic depleted or curtailed the social support that would otherwise protect against negative effects. Social cohesion in our study behaves in a similar fashion, whereby the disaster may be contributing to the deterioration of social capital resources. Evidence from a study of Colombian university students demonstrated a decrease in both cognitive and behavioral social capital between January and August 2020 (51), suggesting that fewer of these resources were available as the pandemic continued and perhaps explaining why social capital, which had been associated with lower levels of depressive symptoms pre-pandemic, was no longer associated with depressive symptoms at the second time point. Although resilience in the present study did not completely deteriorate at higher levels of pandemic impact, its protective effects certainly narrowed. That it did not completely deteriorate may be a result of the relatively small sample size, or it may reflect something about the strength of this particular trait. The decrease in resilience is in line with data from a national study finding that resilience was significantly lower during the pandemic compared to pre-pandemic norms, suggesting that this resource may have also been adversely affected by the pandemic (17). When the effects of the pandemic are more intense, psychosocial resources that were previously beneficial are no longer able to provide the same buffers for mental health.

### 4.3 Racial disparities

The final objective of the study was to describe racial disparities in the association between pandemic impacts and general well-being. Not surprisingly, *Black respondents tended to experience higher levels of pandemic impacts than White respondents*; these differences were particularly marked for work-related impacts, finance-related impacts, and family-related impacts.

#### 4.3.1 Race and social support

*Black respondents in our study also tended to report lower levels of each of the three buffering characteristics of social support, resilience, and social cohesion*. It has been well-documented in the disaster literature that racial/ethnic minorities receive less social support than White persons. For example, a study conducted after Hurricane Hugo in 1989 demonstrated that Black residents in hurricane-affected areas of the southern United States received less tangible support than equally affected White disaster victims, and the disaster exposure sharpened their relative disadvantage (52). Extending the disaster analogy to the COVID-19 pandemic, it is not surprising that these disparities are still playing out.

#### 4.3.2 Race and resilience

In contrast to our finding of less resilience among Black compared to White persons, a national study of US respondents defined resilience as more optimism and better mental health and found that Black persons had higher levels of it than their White counterparts throughout the pandemic (53). Similarly, a study of US women in 2011 reported the highest levels of resilience, measured via the Brief Resilience Scale, among Black women compared to women of other race/ethnicities (32). In Louisiana, by contrast, the Behavioral Risk Factor Surveillance System demonstrated lower scores on the Brief Resilience Scale for Black individuals compared to White individuals in 2022, during the COVID-19 pandemic (33). Yet another study of adults in the New York City area conducted after the September 11 terrorist attack found no significant differences between Black and White persons in resilience scores as defined by the number of PTSD symptoms in the first 6 months after the attack (34). The differences in findings between the current study and what is found in the literature may be due to differences in how resilience was measured in each study, the context (disaster, pandemic, or not), or geographic area (United States, deep South, Northeast). Future research on racial differences in resilience should focus on common definitions and contexts.

#### 4.3.3 Race and social cohesion

Black respondents in our study tended to experience lower levels of social cohesion than White respondents. While there is little agreement in the literature surrounding the effects of race on social cohesion, owing to differing conceptualizations of both social cohesion and race (54), some studies have suggested that Blacks experience lower levels of social cohesion than Whites (36). One study of largely segregated neighborhoods in a Midwestern city, that used the same social cohesion measure as the current study, found that majority Black neighborhoods had lower levels of social cohesion than majority White neighborhoods (55). This difference was explained by residents’ perceptions about the amount of effort required to change undesirable aspects of the neighborhood. It is possible that other neighborhood characteristics as well, such as housing insecurity, could be hindering opportunities for social cohesion. Future avenues of research could explore the specific mechanisms behind the relationships between race and social cohesion.

#### 4.3.4 Race modifies the relationship between pandemic stressors and general well-being

Although no unadjusted relationships were found between race and general well-being in the present study, *race was an important effect modifier of the pandemic-general well-being relationship*.

##### 4.3.4.1 Social support and resilience

For example, both social support and resilience ceased to be protective for general well-being much sooner for Black persons than for White persons. This suggests that the social support deterioration theory mentioned above (50) is particularly salient for Black individuals. While White persons are able to translate high levels of social support and resilience into general well-being regardless of their level of pandemic impact, Black persons show that the buffering ability of these resources is eventually depleted when they are impacted by the pandemic at higher levels. These findings further highlight the deleterious impacts of the pandemic for racial minorities.

##### 4.3.4.2 Social cohesion

With social cohesion, a component of social capital, Black persons tended to fare slightly better than White persons. Black persons with higher levels of social cohesion had higher levels of general well-being across most of the pandemic impact scale, while White persons with higher social cohesion only had better general well-being at the lower ends of the pandemic impact scale. Little research has been conducted on racial differences in social capital, much less how social capital can act as a buffer to mental health during a pandemic. The concepts of bonding, bridging, and linking forms of social capital could be used as a foundation for designing interventions or policies that may mitigate negative pandemic impacts on mental health, though more research is needed to understand how racial differences impact these specific relationships.

### 4.4 Limitations

A few limitations of this study should be noted. (1) It was a cross-sectional study conducted during the first 6 months of the pandemic. It is possible that as the pandemic has continued and evolved, the nature of its impact may have worsened or even started to improve. Future research on different time periods within the pandemic would help elucidate the nature of its impact on mental health. (2) This study was also unable to include data on pre-pandemic mental health. General well-being measured during the pandemic may have been influenced by pre-pandemic well-being, which could have affected results. (3) The EPII, the measurement tool used to measure pandemic impact, is a relatively new instrument with little data available yet on psychometric properties and optimal scoring procedures, and inconsistent use in the specific items retained. Nevertheless, it is a comprehensive instrument with face validity that adds to our understanding of pandemic impacts. (4) The GWB scale, used to measure the outcome of general well-being for the study, asks about respondents’ experiences “during the last month.” It is possible that some recall bias may have occurred in the in respondents’ interpretation of the questions’ time frame.

## 5 Conclusion

The COVID-19 pandemic has impacted mental health in unprecedented ways, prompting research to better understand the pathways through which mental health is affected and how racial disparities might influence these pathways, as Black persons tend to be more impacted by the pandemic than White persons. The psychosocial resources of social support, resilience, and social cohesion are important to consider when creating policies and interventions designed to ameliorate the detrimental effects on mental health during a crisis, but these resources can get compromised as the crisis endures, and this can happen differentially for Blacks compared to Whites. Specifically, social support and resilience appear to deteriorate more rapidly for Black persons compared to White persons, while social cohesion appears to deteriorate more rapidly for White persons compared to Black persons. Future research should consider the specific domains of pandemic impact that affect well-being and tailor interventions around them. This study also highlights the importance of evaluating recovery and mitigation interventions in light of cultural contexts, as an intervention may work well for one sub-population but not for another. Other buffering factors should also be identified and explored, including different personality traits, various components of social capital, and even neighborhood environments. Specific causal pathways and hypotheses involving the relationships that may exist between buffering factors could be elucidated as well. For example, living in neighborhoods high in social cohesion may lead to increased individual resilience, which may in turn lead to improved mental health (35). The long-term effects of pandemic-related stressors are also a critically important area of future research. Results from the present study also have implications for current policy and practice. Mitigation and recovery efforts should consider feasibility of interventions across race/ethnicity categories and include culturally sensitive components to bolster identified buffering factors.

## Data availability statement

The raw data supporting the conclusions of this article will be made available by the authors, without undue reservation.

## Ethics statement

The study involving humans were approved by Louisiana State University Health Sciences Center-New Orleans Institutional Review Board. The studies were conducted in accordance with the local legislation and institutional requirements. The participants provided their written informed consent to participate in this study.

## Author contributions

AR: Conceptualization, Formal analysis, Investigation, Methodology, Project administration, Supervision, Visualization, Writing – original draft, Writing – review & editing. EO: Formal analysis, Methodology, Supervision, Writing – original draft, Writing – review & editing. TP: Data curation, Formal analysis, Writing – original draft, Writing – review & editing. EP: Conceptualization, Funding acquisition, Investigation, Resources, Writing – original draft, Writing – review & editing.
